# The Impact of MRI Features and Observer Confidence on the Treatment Decision-Making for Patients with Untreated Glioma

**DOI:** 10.1038/s41598-019-56333-x

**Published:** 2019-12-27

**Authors:** Paulina Due-Tønnessen, Marco C. Pinho, Kyrre E. Emblem, John K. Hald, Masafumi Kanoto, Andreas Abildgaard, Donatas Sederevicius, Inge R. Groote, Otto Rapalino, Atle Bjørnerud

**Affiliations:** 1 Department of Radiology, Division of Radiology and Nuclear Medicine, Oslo University Hospital, Oslo, Norway; 2 Faculty of Medicine, University of Oslo, Oslo, Norway; 3 Department of Radiology, University of Texas Southwestern Medical Center, Dallas, TX USA; 4 Department of Diagnostic Physics, Division of Radiology and Nuclear Medicine, Oslo University Hospital, Oslo, Norway; 5 Department of Diagnostic Radiology, Faculty of Medicine, Yamagata University, Yamagata, Japan; 6 Department of Radiology and Athinoula A. Martinos Center for Biomedical Imaging, Massachusetts General Hospital and Harvard Medical School, Boston, MA USA; 7 Department of Physics, Faculty of Mathematics and Natural Sciences, University of Oslo, Oslo, Norway

**Keywords:** Diagnostic markers, Predictive markers, Cancer imaging, Cancer therapy

## Abstract

In a blind, dual-center, multi-observer setting, we here identify the pre-treatment radiologic features by Magnetic Resonance Imaging (MRI) associated with subsequent treatment options in patients with glioma. Study included 220 previously untreated adult patients from two institutions (94 + 126 patients) with a histopathologically confirmed diagnosis of glioma after surgery. Using a blind, cross-institutional and randomized setup, four expert neuroradiologists recorded radiologic features, suggested glioma grade and corresponding confidence. The radiologic features were scored using the Visually AcceSAble Rembrandt Images (VASARI) standard. Results were retrospectively compared to patient treatment outcomes. Our findings show that patients receiving a biopsy or a subtotal resection were more likely to have a tumor with pathological MRI-signal (by T2-weighted Fluid-Attenuated Inversion Recovery) crossing the midline (Hazard Ratio; HR = 1.30 [1.21–1.87], *P* < 0.001), and those receiving a biopsy sampling more often had multifocal lesions (HR = 1.30 [1.16–1.64], *P* < 0.001). For low-grade gliomas (N = 50), low observer confidence in the radiographic readings was associated with less chance of a total resection (*P* = 0.002) and correlated with the use of a more comprehensive adjuvant treatment protocol (Spearman = 0.48, *P* < 0.001). This study may serve as a guide to the treating physician by identifying the key radiologic determinants most likely to influence the treatment decision-making process.

## Introduction

Gliomas remain the most common primary brain tumors in adults^1,2^ and are classified according to histopathological features using the World Health Organization (WHO) grading system^3^. While contrast-enhancement Magnetic Resonance Imaging (MRI) is a supplement for glioma characterization and monitoring^4,5^, surgery as the first-line defense makes MRI unlikely to replace histopathology as the benchmark for classification of tumor type and grade. Instead, MRI may better capture the heterogeneity and structural complexity of the disease, and presents an attractive source of complementary information for treatment decisions^6^.

However, which radiologic features are systematically associated with the subsequent patient treatment plan, and moreover, how observer confidence influences the decision-making process are underreported. Performing a study to address these issues is not straightforward because radiologic features of glioma grades, unlike histopathology, do not follow the same level of quantification, but are arguably subjective. Moreover, functional information from diffusion MRI and dynamic susceptibility contrast (DSC)-MRI, provide data that may not fit histopathologic classification nor traditional MRI evaluation. While perfusion MRI may influence a hypothetical management plan^7^, to our knowledge, there is a paucity of literature addressing the extent to which such functional MRI techniques affect treatment decisions in a real clinical setting^8–10^.

To this end, using a large database of clinical and imaging data in a blind, cross-institutional, multi-observer setting, the purpose of our study was to perform a retrospective and unbiased evaluation on the impact of the radiologic assessment and observer confidence of MRI-based preoperative glioma characterization on subsequent treatment decisions.

## Materials and Methods

### Ethical approval and informed consent

Ethical approvals were obtained from institution A (Oslo University Hospital, Oslo, Norway) by the Norwegian Regional Committees for Medical and Health Research Ethics (reference number 2013/81) and for institution B (Massachusetts General Hospital, Boston, MA, USA) by the Partners Human Research Committee (PHRC) Institutional Review Board (2012P000303), respectively. According to the respective national laws, written informed consent was required and obtained for all subjects (patients) from institution A, whereas written informed consent was waived by the Institutional Review Board committee of institution B due to the retrospective nature of the study. All procedures performed in studies involving human participants were in accordance with the ethical standards of the institutional and/or national research committee and with the 1964 Helsinki declaration and its later amendments or comparable ethical standards.

### Patients and treatment options

**Figure 1:**
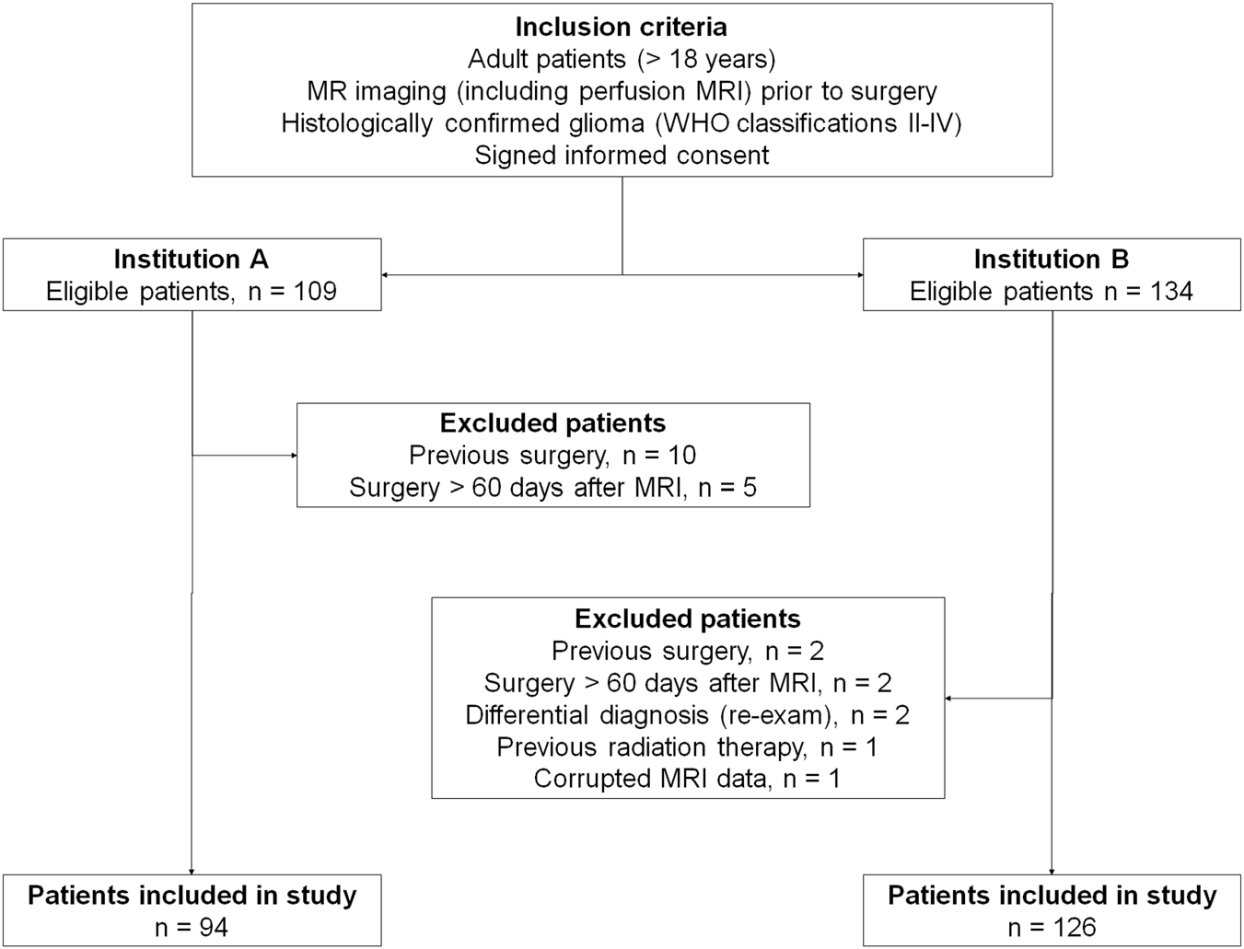
Flow chart of study inclusion criteria. Flow chart showing study inclusion and exclusion criteria. From May 2003 to July 2012, a total of 243 adult patients met the inclusion criteria from both institutions combined. Twenty-three patients were excluded because of undisclosed previous surgery or treatments before the MRI exam (upon re-exam), missing or inconclusive surgery and/or histopathology after the MRI exams, as well as corrupted MRI data. The final study sample for analysis was 220 patients.

We retrospectively included 220 previously untreated adult patients from two institutions on different continents. Of these, 94 patients came from institution A (49 males, median age 49 years, range 23–79 years), and 126 patients came from institution B (69 males, median age 56 years, range 21–87 years). Institution A is a national referral site for brain tumor patients and accounts for almost 50% of all patients in the country. All patients were referred to a diagnostic, contrast-enhanced MR exam between 2003 and 2012, before surgery and subsequent histopathological diagnosis of a WHO grade II–IV glioma (Fig. 1). Patient demographics, histopathological assessments, Karnofsky Performance Status (KPS) at the time of MRI are summarized in Table 1. Per study protocol, treatment options were reviewed up until the end of August 2013 using patient medical records and hospital registry systems. The treatment data included (Table 2); steroid use at the time of MRI, type of surgery (biopsy, subtotal resection <90% or gross total resection >90%), type of adjuvant therapy (fractionized radiotherapy, chemotherapy, combined chemoradiation and/or anti-angiogenic therapy with bevacizumab)^11^, and the number of reoperations (no/one/multiple) within the study period. Post-surgery treatment was decided using all information available to the treating physicians at the time, including MRIs, clinical information and histopathological diagnosis.

**Table 1:** Patient demographics and DSC imaging parameters.

Institution	Vendor and field strength	Imaging parameters	Histology	#patients	WHO grade	Age		Gender	KPS
[#patients]	[number of patients]	[DSC-MRI]				[median]	[range]	[female/male]	%
**A** [94]	Siemens 1.5T Avanto [42], Sonata [6], Symphony [46]	Axial 12–14 slices; single-shot GE EPI; 50–80 volumes; TR = 1400–1590 ms; TE = 30–52 ms; voxel size 1.8 × 1.8 × 5 mm^3^; slice spacing 1.5 mm; FA = 90°; 0.2 mmol/kg Gd-DTPA (Gadovist, Bayer Pharma AG)	DA	17	II	41	23–63	7/10	90–100
			OA	6	II	40	26–70	3/3	100
			OD	5	II	36	30–64	4/1	90–100
			GA	3	II	52	41–69	1/2	100
			AA	7	III	68	35–79	5/2	80–100
			AOA	5	III	62	28–82	2/3	90–100
			AOD	2	III	40	28–53	0/2	100
			GBM	49	IV	60	40–78	23/26	50–100
**B** [126]	Siemens 1.5T Avanto [3]; Siemens 3T TimTrio [16]; GE 1.5T Signa HDxt [107]	Axial 14–16 slices; single-shot GE/SE EPI; 80 volumes; TR = 1400–1500 ms; TE = 20–40/100 ms; voxel size 1.9 × 1.9 × 5 mm^3^; slice spacing 1 mm; FA = 60°; 0.1–0.2 mmol/kg Gd-DTPA (Magnevist, Bayer Pharma AG)	DA	8	II	39	24–62	5/3	90–100
			OA	8	II	39	26–55	3/5	90–100
			OD	1	II	45	45	0/1	90
			EP	1	II	68	68	0/1	100
			CG	1	II	53	53	1/0	80
			AA	24	III	49	26–87	10/14	60–100
			AOA	9	III	40	21 74	4/5	90–100
			AOD	2	III	58	37–78	0/2	70–90
			GBM	72	IV	64	33–87	34/38	50–100

Note. WHO = World Health Organization, KPS = Karnofsky performance status, GE = gradient echo, SE = spin echo, EPI = echo planar imaging,

TR = repetition time, TE = echo time, FA = flip angle, Gd-DTPA = gadopentetate-dimeglumine, DA = Diffuse Astrocytoma, OA = Oligoastrocytoma,

OD = Oligodendroglioma, EP = Ependymoma, CG = Chordoid glioma, AA = Anaplastic Astrocytoma, AOA = Anaplastic Oligoastrocytoma,

AOD = Anaplastic Oligodendroglioma, GBM = Glioblastoma Multiforme.

**Table 2:** Histopathologic diagnoses and treatments used in the study.

Institution	Histology	#patients	WHO grade	Steroids at MRI	Resection	Reoperation	Adjuvant Therapy
[#patients]				Yes/No	B/<90%/>90%	Yes/No	C/R/CR
**A** [94]	DA	17	II	7/10	7/8/2	5/17	3/2/6
	OA	6	II	2/4	1/3/2	1/6	1/0/1
	OD	5	II	2/3	2/3/0	1/5	3/0/1
	GA	3	II	1/2	1/2/0	0/3	0/1/1
	AA	7	III	4/3	3/3/1	0/7	0/3/4
	AOA	5	III	2/3	0/4/1	2/5	0/1/4
	AOD	2	III	1/1	0/0/2	0/2	0/1/1
	GBM	49	IV	31/18	11/28/10	7/49	1/3/36
**B** [126]	DA	8	II	0/8	4/2/2	0/7^†^	0/3/2
	OA	8	II	0/8	1/0/7	0/8	0/0/3^*^
	OD	1	II	0/1	0/0/1	0/1	0/1/0
	EP	1	II	0/1	0/0/1	0/0^†^	0/0/0
	CG	1	II	0/1	0/1/0	0/1	0/1/0
	AA	24	III	4/20	14/6/3^†^	1/21^†^	0/2/18^*†^
	AOA	9	III	1/8	1/7/1	2/7	0/2/6^*^
	AOD	2	III	1/1	0/1/1	1/1^†^	0/0/2^*^
	GBM	72	IV	16/56	20/32/20	7/61^†^	0/5/56^*†^

Note. WHO = World Health Organization, B = Biopsy, <90% = subtotal resection, >90% = gross total resection.

C = Chemotherapy, R = Radiotherapy, CR = Chemoradiation, DA = Diffuse Astrocytoma, OA = Oligoastrocytoma.

OD = Oligodendroglioma, EP = Ependymoma, CG = Chordoid glioma, AA = Anaplastic Astrocytoma.

AOA = Anaplastic Oligoastrocytoma, AOD = Anaplastic Oligodendroglioma, GBM = Glioblastoma Multiforme.

†incomplete data, *some patients receiving CR also received concomitant anti-angiogenic therapy.

### Histopathologic analyses

Tumor tissue from needle biopsies or surgical resections were routinely formalin-fixed and paraffin-embedded before diagnostic review by an experienced neuropathologist. Because of the retrospective nature of our study and the closure date of August 2013, all tumors were classified according to the WHO 2007 criteria (glioma grades II–IV). Only previously untreated patients with a newly diagnosed glioma by histopathology were included in the study.

### MR imaging

Imaging was performed with 1.5 Siemens scanners at institution A, and 3 Tesla Siemens and 1.5 Tesla GE (General Electric) scanners at institution B, with 8-, 12-, or 32-channels (Siemens) and 8-, or 16-channels (GE) head coils (Table 1). An important aspect of study protocol was the use of actual clinical data without any enforced cross-institutional standardization of imaging protocols.

For institution A, a standard imaging protocol^12^ included 0.45 × 0.45 × 5mm^3^ voxel size two-dimensional axial 19-slice fast spin-echo T_2_-weighted (repetition time TR = 4000 ms, echo time TE = 104 ms), coronal 25-slice T2-weighted fluid-attenuated inversion recovery (FLAIR) (TR = 9050 ms, TE = 114 ms; inversion time TI = 1500 ms) and 0.45 × 0.45 × 5 mm^3^ voxel size axial/coronal/sagittal 19-slice pre/post-contrast spin-echo T_1_-weighted (TR = 500 ms, TE = 7.7 ms) images. Diffusion MRI was available in 83 of 94 subjects using axial 19-slice single-shot, spin-echo echo-planar imaging with TR = 3300 ms, TE = 95 ms, 1.8 × 1.8 × 5mm^3^ voxel size, three averages, 1.5 mm inter-slice gap, 90° flip angle and b-values of 0, 500, 1000 s/mm^2^ in three orthogonal directions. DSC-MRI was performed in all patients (sequence details shown in Table 1), including administration of a single dose of 0.2 mmol/kg gadobutrol (Gadovist®, Bayer Schering Pharma, Berlin, Germany) at a rate of 5 mL/sec followed by a 20 ml saline flush (BB Melsungen, Melsungen, Germany).

For institution B, the imaging protocol^13^ mirrored that of institution A, including concurrent diffusion MRI in 119 of 126 patients. All patients had DSC-MRI with either a single or a double dose of 0.1 mmol/kg gadopentate dimeglumine (Magnevist®, Bayer Schering Pharma, Berlin, Germany) at a rate of 5 mL/sec followed by 20 ml saline (sequence details shown in Table 1).

### Image pre-processing

All image pre-processing was performed using nordicICE (NordicNeuroLab AS, Bergen, Norway) or the open source platform 3D Slicer. As previously described^14^, a board-certified neuroradiologist at each institution identified tumor regions on conventional MRIs using manual outlining in nordicICE for institution A, and a semi-automatic approach in 3D Slicer^15^ for institution B. Using nordicICE, apparent diffusion coefficient (ADC) maps were estimated using Stejskal-Tanner diffusion approximation^16^ and DSC-MRI data were automatically processed to create relative cerebral blood volume (rCBV) maps using standard kinetic modelling and corrected for contrast agent extravasation^17^. All rCBV maps were normalized to normal-appearing tissue^18^ and presented as semi-transparent color overlays on anatomical MRIs.

### Observers

Two neuroradiologists with >10 and >15 years of clinical experience with brain MRI, were included from institution A. Two additional neuroradiologists were included to represent institution B, one with >10 years of clinical experience with brain MRI and working at institution B. The other observer representing institution B was a consultant neuroradiologist from a third institution with >5 years of clinical experience with brain MRI. The observers from institution A reviewed the MRI data of institution B, and vice versa.

### First MRI reading

Using anatomical and diffusion MRI only, the observers recorded their scores from visual grading using the Visually AcceSAble Rembrandt Images (VASARI) standardized feature set^6^ through the joint National Institute of Health (NIH) and National Cancer Institute (NCI) initiated Annotation and Image Markup (AIM) scoring template for gliomas. The VASARI scoring system includes 19 semantic descriptors of imaging features of brain tumors (Supplementary Table 1). Tumor location was also assessed by inclusion (Yes/No) of the parietal, frontal, occipital and temporal lobes, cerebellum, the basal ganglia, thalamus, insula, as well as deep white matter involvement including corpus callosum and internal capsule.

The observers then classified the gliomas per suggested WHO grade, and the corresponding level of confidence using a four-level classification scheme; (**I**) doubtful (<50% certainty), (**II**) somewhat confident (50–70% certainty), (**III**) very confident (70–90% certainty), and (**IV**) extremely confident (>90% certainty). To mimic a real-world clinical situation, patient age and the presenting neurologic symptoms as written on the admission recording were available to the observer, while all other information was blind.

### Second reading

After an interval of at least one month, the observers were again presented with the MRI data to perform a second, repeated reading, with a re-shuffled patient sequence. The two readings where averaged to compensate for intra-observer variability. Immediately after the second reading, the observers were also presented with DSC-MRI data. The observers then re-examined all available imaging data using rCBV color maps at will, and recorded a third set of glioma grades and confidence levels.

### Statistical analyses

Any institutional differences in patient demographics and MRI findings were assessed using independent samples two-sided t-tests. Treatment endpoints were tested for associations by stepwise linear regression models (ANOVA), or rank tests (Mann-Whitney or Kruskal-Wallis) and Spearman correlation if parametric assumptions were not met. Model inputs included patient gender, age at time of MRI (years), KPS (%), total tumor volume and edema volume (cubic centimeter), and treatment options. The stepwise linear model was halted at the first value not passing significance.

Intra-observer reproducibility was assessed by glioma grade and observer confidence using intra-class correlation coefficients (ICC) between the first and second readings of the conventional MRIs only (adding rCBV refutes the test-retest scheme). Moreover, to overcome dependencies to observer- and institutional variations, the data were also pooled into a single, 220 patient cohort. A patient was labeled according to the average score between the first- and second observer readings, and thereafter across two observers at the same institution. For missing data by one or more observers, the recorded value of the remaining observer was used.

Statistical analyses were performed using SPSS 22 (SPSS Inc., USA). A *P*-value of 0.05 was considered significant, and all tests with multiple comparisons were Bonferroni corrected (*P* = 0.05/number of tests).

## Results

### Institutional differences in patient data and treatment options

The ratio of low-grade to high-grade gliomas was significantly higher for institution A compared to institution B (49% versus 18%, *P* = 0.003). However, the distributions of WHO grades within the institution was similar (Table 1; *P* = 0.047; not passing Bonferroni). The average KPS was correspondingly higher at institution A (90.53% ± 11.20% versus 86.59% ± 9.48%, *P* = 0.005), and steroids during initial MRI were administered more frequent (53% versus 17%, *P* < 0.001). The number of gross-total resections in WHO grade II gliomas was lower for institution A compared to institution B (Table 2; 13% versus 58%, *P* = 0.018). Patients at institution A were also more likely to have repeated surgery (all patients: 26% versus 9%, *P* = 0.002), owing to higher repeated surgery of WHO grade II gliomas (42% versus 0%, *P* < 0.001). Chemotherapy as a monotherapy was only administered at Institution A, while anti-angiogenic drugs were only administered at institution B. All patients on anti-angiogenic therapy also received combined chemo-radiation.

### Observer outcome measures and intra-observer repeatability

An overview of the average WHO grade and corresponding confidence levels of all observers are shown in Table 3. At both institutions and for all observers, the proposed WHO grades did not change over the course of the study readings. In contrast, the confidence scores increased significantly for all observers with the addition of the rCBV map (at the *P* < 0.001 level, all observers, Table 3). Moreover, for institution A, the ICCs when grading patients by WHO grades II-IV were 0.93 (*P* < 0.001, n = 91) and 0.88 (*P* < 0.001, n = 73) for the two observers, respectively. The ICCs of the corresponding confidence scores were lower at 0.7381 (*P* < 0.001, n = 91) and 0.58 (*P* < 0.001, n = 73), respectively. For institution B, the ICCs when grading patients by WHO grades II-IV were 0.69 (*P* < 0.001, n = 120) and 0.83 (*P* < 0.001, n = 124) for observer 1 and 2, respectively. Again, the ICCs of the corresponding confidence scores were lower at 0.35 (*P* < 0.001, n = 120) and 0.25 (*P* = 0.002, n = 124) for observer 1 and 2, respectively.

**Table 3:** Resulting WHO grading and confidence scores.

Observer [MRI data from]	MRI #1 [WHO grade]^†^ *[confidence]*^†^	MRI #2 [WHO grade] [*confidence*]	MRI #2 +CBV[WHO grade] [*confidence*]
**Observer 1** (>10yrs) [Institution A data]	**3.48** ± **0.70 (n** = **93)** *2.92* ± *0.99 (n* = *86)*	**3.48** ± **0.70 (n** = **91)** *3.01* ± *1.07 (n* = *83)*	**3.54** ± **0.69 (n** = **91)** *3.35* ± *0.85 (n* = *89)*^***A,B***^
**Observer 2** (>5yrs) [Institution A data]	**3.26** ± **0.80 (n** = **85)** *2.69* ± *0.85 (n* = *82)*	**3.24** ± **0.82 (n** = **82)** *2.70* ± *0.78 (n* = *81)*	**3.29** ± **0.81 (n** = **82)** *3.10* ± *0.75 (n* = *81)*^***A,B***^
**Observer 1** (>15yrs) [Institution B data]	**3.36** ± **0.80 (n** = **120)** *2.26* ± *0.65 (n* = *111)*	**3.26** ± **0.88 (n** = **121)** *2.08* ± *0.59 (n* = *105)*	**3.26** ± **0.89 (n** = **121)** *2.72* ± *0.65 (n* = *119)*^***A,B***^
**Observer 2** (>10yrs) [Institution B data]	**3.44** ± **0.77 (n** = **121)** *2.83* ± *0.89 (n* = *117)*	**3.33** ± **0.84 (n** = **126)** *2.29* ± *0.66 (n* = *114)*^***A***^	**3.32** ± **0.84 (n** = **126)** *2.62* ± *0.74 (n* = *118)*^***B***^

Note. ^†^Data show average values (unitless), standard deviations and sample size.

**A** = different from MRI #1 at the *P* < *0.001* level, **B** = different from MRI #2 at the *P* < *0.001* level, (>5–15 yrs) corresponds to years of clinical experience with brain MRI.

WHO = World Health Organization, MRI #1 = first conventional MRI reading,

MRI #2 = second conventional MRI reading, +CBV = with addition of CBV maps.

### Institutional differences in MRI features

The average size of the tumors was smaller (37.63 ± 33.34 mL versus 68.42 ± 58.87 mL, *P* < 0.001), while the average peritumoral edema region was larger (53.43 ± 47.01 mL versus 34.38 ± 50.90 mL, *P* = 0.012) at institution A compared to institution B. Matched for WHO glioma grade, ependymal extension was the only imaging features from the first MRI reading separating patients of institution A from institution B (67% versus 17%, *P* < 0.001). For WHO grades II and IV only, the non-enhancing tumor margins were less well-defined at institution A compared to institution B (30% versus 73%, *P* < 0.003). There was no significant difference in the enhancement quality between 1.5 and 3 Tesla systems (Table 1) (*P* > 0.79; both observers), nor between a single - versus double-dose contrast agent administration (*P* > 0.79).

### Associations between MRI features and subsequent neurosurgery

Table 4 highlights the pan-institutional imaging features associated with the choice of surgical procedure. In short, by VASARI, patients with a biopsy or a subtotal resection were more likely to have a tumor with pathologic FLAIR/T2 signal crossing the midline, and those receiving a biopsy sampling more often had multifocal or multicentric lesions. Patients with low post-contrast enhancement quality (0–1) had longer time between the pre-surgical MRI and subsequent surgery (trimmed mean = 10.84days versus 3.92days, *P* < 0.01, Hazard Ratio [95% conf.int]; HR = 1.21 [1.06–1.38]). For WHO grade IV glioblastomas only (N = 121), lack of FLAIR/T2 signal crossing the midline and no satellite enhancement foci were associated with a gross total resection (*P* < 0.001, HR = 1.37 [1.17–1.61]).

**Table 4:** Associations between MRI features and subsequent neurosurgery (both institutions).

MRI feature^#^	Biopsy	Subtotal resection	Gross total resection	Repeated surgery		Hazard ratio
	±st.dev	±st.dev	±st.dev	Yes	No	[95% conf.int]
FLAIR/T2 signal cross midline	**45% (29/64)**	30% (30/99)	6% (3/51)	—	—	1.30 [1.21–1.87] ***
Enhancement quality	1.96 ± 1.17	**2.77** ± **0.90**	1.97 ± 1.31	—	—	1.27 [1.11–1.56] ***
Multifocal lesions	**42% (27/64)**	27% (27/99)	6% (3/51)	—	—	1.30 [1.16–1.64] ***
Pial invasion	—	—	—	**43% (16/36)**	17% (31/179)	1.30 [1.11–1.48] **
Faciliated diffusion	—	—	—	**1.52** ± **0.40**	1.70 ± 0.61	1.32 [1.09–1.37] **

Note. ***Significant features at the *P* < 0.001 level (Bonferroni corrected).

**Significant features at the P < 0.01 level (Bonferroni corrected).

^**#**^Observer scorings deemed indeterminate were excluded from analysis.

Highest incidence/value highlighted in bold.

For Institution A, patients with a biopsy sampling or a subtotal resection more often had tumors with an infiltrative T1/FLAIR ratio (1.94 ± 0.71 and 1.91 ± 0.68 versus 1.41 ± 0.54, *P* = 0.008) and less pial invasion (16% and 41% versus 53%, *P* = 0.002) compared to those with a total resection. Findings of institution B mirrored the pan-institution analyses (Table 4).

Finally, repeated surgery at both institutions was associated with a higher incidence of pial invasion (67% versus 38%, *P* = 0.001) and less restricted diffusion (1.52 ± 0.40 versus 1.70 ± 0.61, *P* = 0.002). For WHO grade IV glioblastomas, more peritumoral edema (74.94 ± 83.93 mL versus 44.71 ± 38.95 mL, *P* = 0.001) and lack of satellite enhancement foci (24% versus 43%, *P* = 0.001) were also associated with repeated surgery (N = 17 of 121).

### Associations between MRI features and adjuvant therapy

Table 5 summarizes the associations relevant for both institutions. In short, patients receiving steroids at the time of MRI (N = 72) more frequently had tumors with poorly defined non-enhancing margins (65% versus 40%, *P* < 0.001), and pial invasion (34% versus 16%, *P* < 0.001). This association also included ependymal extension, but it did not pass Bonferroni correction. Cortical involvement was seen less frequent in patients receiving steroids (74% versus 79%, *P* = 0.002). For patients receiving mono-radiotherapy (N = 24), a lower WHO glioma grade when including DSC-MRI was the only associated variable (2.96 ± 0.81 versus 3.49 ± 0.74, *P* = 0.001). For patients receiving chemotherapy only (Institution A; N = 8), the T1/FLAIR ratio was more infiltrative (2.06 ± 0.73 versus 1.52 ± 0.61, *P* = 0.027; Mann-Whitney) and the suggested grade with DSC-MRI was lower (2.88 ± 0.64 versus 3.46 ± 0.76, *P* = 0.014; Mann-Whitney). Patients receiving combined chemo-radiation therapy (N = 141) had a higher suggested WHO grade with DSC-MRI (3.64 ± 0.64 versus 3.06 ± 0.84, *P* < 0.001) and lack of deep-brain involvement by less ependymal extension (34% versus 48%, *P* = 0.001) and less involvement of the basal ganglia (24% versus 36%, *P* = 0.002). For WHO grade IV glioblastomas, tumor with less frequent involvement of the basal ganglia (23% versus 54%, *P* < 0.001) and more well-defined non-enhancing tumor margins (61% versus 35%, *P* = 0.002) more often received combined chemo-radiation therapy.

**Table 5:** Associations between MRI features and adjuvant therapy (both institutions).

MRI feature^#^	Steroids at MRI		Radiation		Chemo-radiation		Hazard ratio
	Yes	No	Yes	No	Yes	No	[95% conf.int]
Poor non-enhancing margins	**65% (46/70)**	40% (59/145)					1.36 [1.19–1.66]***
Pial invasion	**34% (24/70)**	16% (23/145)					1.35 [1.18–1.71]***
Cortical involvement	74% (52/70)	**79% (115/145)**					1.29 [1.13–1.70]**
Glioma grade (with DSC-MRI)			2.96 ± 0.81	**3.49** ± **0.74**			1.29 [1.05–1.23]**
Glioma grade (with DSC-MRI)					**3.64** ± **0.64**	3.06 ± 0.84	1.38 [1.15–1.45]***
Ependymal extension					34% (47/141)	**48% (38/79)**	1.30 [1.12–1.51]***
Involvement of basal ganglia					24% (34/141)	**36% (29/79)**	1.26 [1.09–1.45]**

Note. ***Significant features at the *P* < 0.001 level (Bonferroni corrected).

**Significant features at the P < 0.01 level (Bonferroni corrected).

^**#**^Observer scorings deemed indeterminate were excluded from analysis.

Highest incidence/value highlighted in bold.

Comparing patients on combined chemo-radiation with- or without additional anti-angiogenic therapy (institution B), patients receiving bevacizumab (N = 29) were more likely to have a tumor in the deep-brain by more frequent involvement of the thalamus (28% versus 11%, *P* = 0.002) and less frequent involvement of the temporal lobes (31% versus 56%, *P* = 0.002).

### Associations between observer confidence and treatment

**Figure 2:**
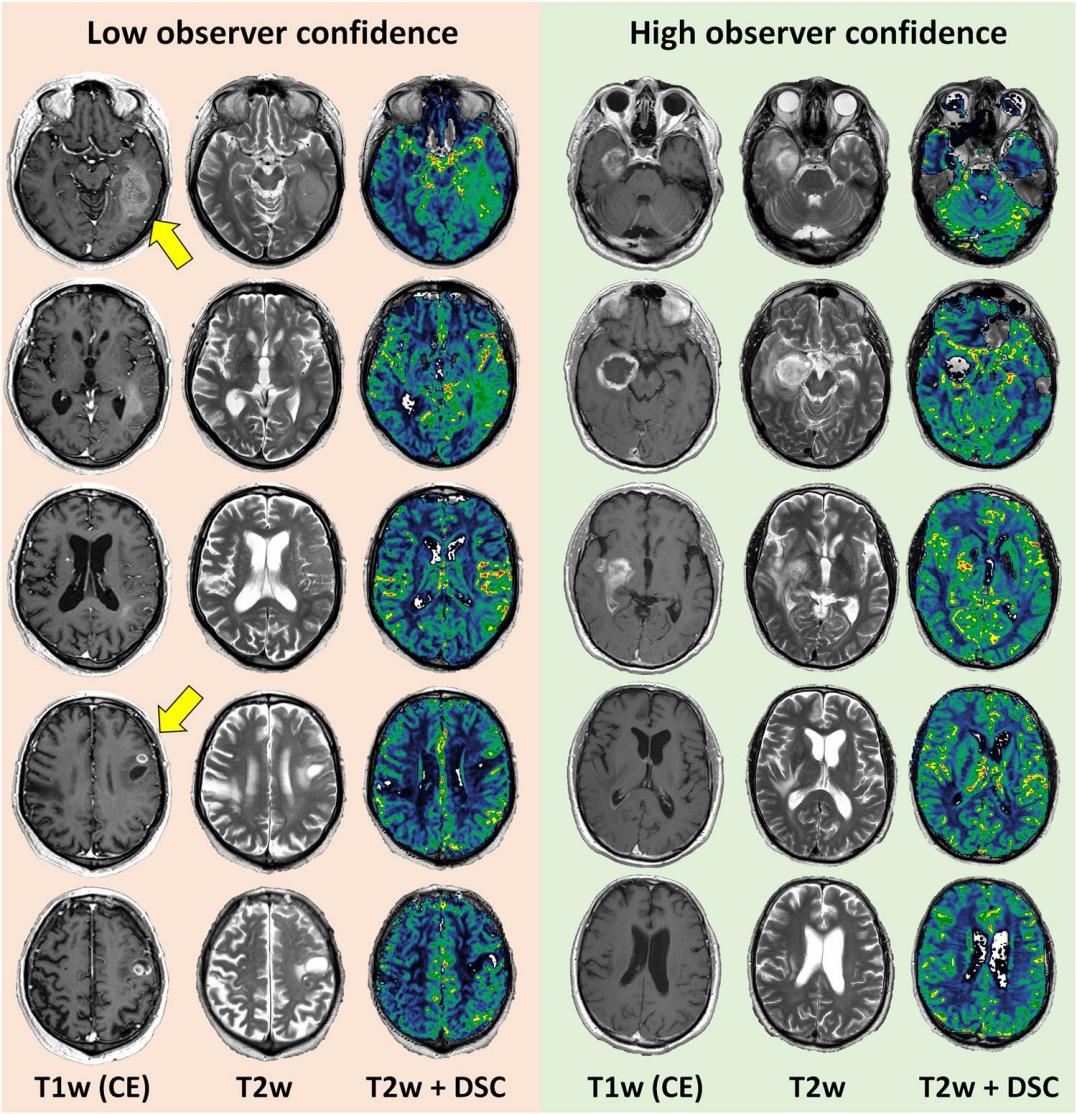
Example MRIs returning low- and high observer confidence. Illustration showing representative contrast-enhanced (CE) T1-weighted (T1w) and T2-weighted (T2w) MRIs and rCBV maps from DSC of two patients from institution A with WHO grade IV glioblastomas. (Left) MRIs of a 74-year old male returning low average observer confidence (‘somewhat confident’, 50–70% certainty), and typical appearance of a multifocal lesion with complex and diffuse contrast-agent enhancement patterns. (Right) MRIs of a 60-year old male returning high average observer confidence (‘extremely confident’, >90% certainty), and with typical appearance of a single lesion with well-defined contrast-enhancement patterns. Adding DSC-MRI reduced the number of VASARI features associated with low observer confidence at the P < 0.001 level. Both patients used steroids at the time of the MRI exam.

Figure 2 shows representative MRI of two patients with low- and high observer confidence, respectively. Steroid use at the time of MRI (N = 72) was associated with higher observer confidence when evaluating anatomical MRI only (2.79 ± 0.75 versus 2.43 ± 0.64, *P* < 0.001). Interestingly, when adding DSC-MRI, the confidence level for the non-steroid group (N = 148) increased significantly (from 2.43 ± 0.64 to 3.03 ± 0.80, paired t-test: *P* < 0.001), and to a higher level than the steroid group (3.03 ± 0.80 versus 2.79 ± 0.75, *P* < 0.001). Moreover, adding DSC-MRI reduced the number of VASARI features associated with low confidence (score 1–2) at the *P* < 0.001 level (median 3 versus 0 features; Supplementary Table 1). For WHO glioma grades II and III (N = 99), observer confidence was associated with the choice of surgical procedure. The lowest observer confidence was found in patients receiving a biopsy only (2.02 ± 0.44) compared to subtotal resection (2.25 ± 0.50) and gross total resection (2.40 ± 0.44, *P* = 0.002).

Finally, for low-grade gliomas (N = 50), the use of adjuvant therapy was inversely correlated with observer confidence (Spearman = −0.48, *P* < 0.001). Patients not receiving any adjuvant therapy had the highest observer confidence (median 2.50, range 2.00–3.25, N = 22), whereas patients on combined chemo-radiation with- or without anti-angiogenic therapy had the lowest observer confidence (median 2.00, range 1.75–3.5, N = 13).

## Discussion

In a blind and retrospective, dual-center, multi-observer study, we quantify pre-treatment radiologic features by MRI that are systematically associated with the outcome of subsequent treatment of adult patients with gliomas and therefore likely to play a key role in the clinical decision-making process. The choice of surgical intervention was associated with the complexity of tumor infiltration, and patients whose tumors had indiscernible contrast-enhancement patterns received a more conservative management by approximately 3 times longer time between the MRI exam and subsequent surgery. Tumors with a pathologic MRI-signal crossing the midline and/or multifocal disease were more likely to have sub-total resection or especially a biopsy. This finding was also associated with lower observer confidence. Tumor progression and the need for repeated surgery where associated with pial invasion and a more edematous signature of the peritumoral region^19–21^. Moreover, steroids were more often seen in invasive and infiltrative tumors with poorly defined non-enhancing margins. Still, use of steroids was also linked to higher observer confidence, where the tumors’ overall appearance probably showed less ambiguous imaging features. For adjuvant therapy and corrected for grade, patients not receiving any additional therapy outside surgery had the highest observer confidence. Observer confidence also returned lower ICCs than those from glioma grading. Interestingly, this suggests that treating physicians are more likely to opt for additional adjuvant treatment options for tumors in which the imaging appearance is less typical, irrespective of histological grade. This fact is not entirely unexpected and likely reflects physicians understanding about the inherent limitations of the WHO grading system as the main or sole determinant of treatment decisions, a historical dogma which is being challenged and replaced by recent discoveries in glioma oncogenetics^22,23^. This finding may also warrant the need for standardized disease registries in order to learn from the decisions made and the subsequent outcomes of previous decision-making processes. Patients receiving combined chemo-radiation had well-defined non-enhancing tumor margins and lack of deep-brain involvement. Because the choice of treatment is arguably linked to glioma location^12,19,24^, these imaging features probably helped identify the target area for radiotherapy. Also, the lack of deep-brain involvement is consistent with the goal of minimizing radiation damage to basic functions of the brain. Instead, patients with tumors of the deep-brain where more likely to receive anti-angiogenic therapy.

Our study advocates the need for a high-quality, focused MRI protocol to complement the clinical and histopathologic data for pre-treatment assessment of patients with brain tumors. Novel imaging techniques are regularly introduced into oncologic research, with the ability to visualize new aspects of tumor pathophysiology, cellularity, metabolic profile and hemodynamic status^4,6,9,25,26^. Glioma imaging protocols are therefore becoming increasingly comprehensive, time-consuming and costly, whilst quantification of any added impact on the decision-making process is still rarely performed. It can therefore be debated under what circumstances the diagnostic process is really improved in a cost-effective way by increasing the number of exams^27^. In line with previous reports, adding DSC-MRI to a conventional imaging protocol improved the observers’ confidence of the glioma characterization in untreated (non-steroid) patients^10^. Also, with DSC-MRI, observers suggesting a lower glioma grade was associated with adjuvant radiation- or chemo- monotherapy, whereas the suggestion of a higher glioma grade was associated with combined chemo-radiation. The reduced number of VASARI features associated with a low observer confidence could potentially be seen as a time-saving feature of DSC-MRI. While comparing subjective confidence scores across that observers with various levels of experience should be performed with care, our results indicate DSC-MRI may aid less experienced readers. For prospective studies, introducing machine learning alternatives may help confirm or identify other relevant imaging feature of the disease, and also reduce the inherent observer variations that follow complex diagnostic readings^13,28^. By comparing the results of an artificial intelligence (AI) model to that of our current expert radiologic examination, we can reveal the added value of the AI model for assessment of disease. Finally, use of AI-based model interpretability may help generate more powerful radiomics signatures from the hidden layers of the neural network beyond the radiologist-labeled, classical VASARI features^29^.

### Our study has some limitations

Owing to inherent regional and national determinants, differences in patient demographics between the two institutions may have influenced our results. However, this difference is also welcomed in what makes a multi-center study stand out from a single-institution analogue, and introduce a compelling range in our findings beyond a certain demographic setting. Moreover, while taking measures to blind the observers, a study design of this nature will never truly mimic the dynamic and complex workup of oncologic practice. Undoubtedly, the treating physician will also include information from the histopathologic analyses in the treatment decision-making process. Therefore, our findings also include imaging analyses from patients of the same WHO type and grade. Also, owing to the retrospective nature of our study, the WHO grading system of 2007 was used in histopathological diagnosis^3^, and neither we, nor the treating physicians at the time, had access to the molecular profiles of individual tumors. Both mutation status of isocitrate dehydrogenase (IDH) 1/2 and methylated O6-methylguanine DNA methyltransferase (MGMT) may influence the treatment decision process, and also potentially be determined by DSC-MRI^26,30^. Furthermore, and unlike today, not all of the anatomical MRI data at the time used a 3D image readout. However, this should only to a limited extent affected the VASARI criteria as presented in our study.

To conclude, in a comprehensive study we identify the key radiographic determinants of glioma patients associated with the treatment decision-making process. The choice of surgical intervention was associated with the complexity of tumor infiltration and low observer confidence was associated with a more extensive adjuvant treatment protocol.

## Supplementary information

Supplementary information 

## Data Availability

The authors declare that all data supporting the findings of this study are available within the paper and its Supplementary Information. The datasets generated during and/or analyzed during the current study are available from the corresponding author on reasonable request.
