# Author Correction: The Impact of MRI Features and Observer Confidence on the Treatment Decision-Making for Patients with Untreated Glioma

**DOI:** 10.1038/s41598-021-99776-x

**Published:** 2021-10-07

**Authors:** Paulina Due-Tønnessen, Marco C. Pinho, Kyrre E. Emblem, John K. Hald, Masafumi Kanoto, Andreas Abildgaard, Donatas Sederevicius, Inge R. Groote, Otto Rapalino, Atle Bjørnerud

**Affiliations:** 1 Department of Radiology, Division of Radiology and Nuclear Medicine, Oslo University Hospital, Oslo, Norway; 2 Faculty of Medicine, University of Oslo, Oslo, Norway; 3 Department of Radiology, University of Texas Southwestern Medical Center, Dallas, TX USA; 4 Department of Diagnostic Physics, Division of Radiology and Nuclear Medicine, Oslo University Hospital, Oslo, Norway; 5 Department of Diagnostic Radiology, Faculty of Medicine, Yamagata University, Yamagata, Japan; 6 Department of Radiology and Athinoula A. Martinos Center for Biomedical Imaging, Massachusetts General Hospital and Harvard Medical School, Boston, MA USA; 7 Department of Physics, Faculty of Mathematics and Natural Sciences, University of Oslo, Oslo, Norway

Correction to: *Scientific Reports*: 10.1038/s41598-019-56333-x, published online 27 December 2019

The original version of this Article omitted an affiliation for Paulina Due-Tønnessen. The correct affiliations are listed below.

Department of Radiology, Division of Radiology and Nuclear Medicine, Oslo University Hospital, Oslo, Norway

Faculty of Medicine, University of Oslo, Oslo, Norway

The original Article and accompanying Supplementary Information file have been corrected.

