# Supplementary material for: The Impact of MRI Features and Observer Confidence on the Treatment Decision-Making for Patients with Untreated Glioma
Source: Sci Rep. 2019 Dec 27;9:19898. doi: 10.1038/s41598-019-56333-x (PMC6934740; doi:10.1038/s41598-019-56333-x)
Supplement: Supplementary file 1 — Supplementary information [file 41598_2019_56333_MOESM1_ESM.docx]

**Supplementary Information for manuscript:**

**The Impact of MRI Features and Observer Confidence on the Treatment Decision-Making for Patients with Untreated Glioma**

Paulina Due-Tønnessen^1,2*^, Marco C. Pinho^3^, Kyrre E. Emblem^4^, John K. Hald^1^, Masafumi Kanoto^5^, Andreas Abildgaard^1^, Donatas Sederevicius^4^, Inge R. Groote^4^, Otto Rapalino^6^ & Atle Bjørnerud^4,7^

1. Department of Radiology, Division of Radiology and Nuclear Medicine, Oslo University Hospital, Oslo, Norway

2. Faculty of Medicine, University of Oslo, Oslo, Norway

3. Department of Radiology, University of Texas Southwestern Medical Center, Dallas, TX, USA

4. Department of Diagnostic Physics, Division of Radiology and Nuclear Medicine, Oslo University Hospital, Oslo, Norway

5. Department of Diagnostic Radiology, Faculty of Medicine, Yamagata University, Yamagata, Japan

6. Department of Radiology and Athinoula A. Martinos Center for Biomedical Imaging, Massachusetts General Hospital and Harvard Medical School, Boston, MA, USA

7. Department of Physics, Faculty of Mathematics and Natural Sciences, University of Oslo, Oslo, Norway

**Online supplementary Table 1**
